# A scoping review of measurement tools and a content validity evaluation of the University of Jyvaskyla Active Aging Scale (UJACAS)

**DOI:** 10.1007/s10433-025-00893-7

**Published:** 2025-11-04

**Authors:** Rosa Napoletano, Antonella Lopez, Sergio Traficante, Elisabetta Ricciardi, Luigi Tinella, Alessandro Oronzo Caffò, Andrea Bosco, Giuseppina Spano

**Affiliations:** 1 Department of Educational Sciences, Psychology, Communication, University of Bari, 70122 Bari, Italy; 2 Department of Humanities, Social Science, and Education, University of Molise, Via Francesco De Sanctis, 86100 Campobasso, Italy; 3 Department of Precision and Regenerative Medicine and Ionian Area, University of Bari, 70124 Bari, Italy; 4 Department of Humanities, Philosophy and Education, University of Salerno, 84084 Salerno, Italy; 5 Department of Psychology and Health Science, Pegaso University, 80013 Naples, Italy

**Keywords:** Active aging, Measurement tool, Questionnaire, Older people, Scoping review

## Abstract

The concept of active aging (AA) was defined as the process of optimizing opportunity for health, security, and social participation for older people. While several questionnaires were developed to measure AA, there is no universal consensus on how to measure it, underlining a lack of clarity about what aspects to consider in the evaluation. To contribute to fill this gap, the aims of these two studies were to provide a guide for aging care professionals to choose the most appropriate questionnaire(s) based on a scoping review of the specialist literature and on a content analysis of the questionnaire, that is, the University of Jyvaskyla Active Aging Scale (UJACAS). Preferred Reporting Items for Systematic reviews and Meta-Analyses extension for Scoping Reviews (PRISMA-ScR) guidelines were used. We found 25 records introducing questionnaires. The content validity evaluation of the most complete questionnaire (in terms of inspected content areas), i.e., UJACAS, was conducted to obtain a complete framework of evaluated aspects. Among the selected questionnaires, heterogeneity was found regarding investigated aspects and relative terms used to describe it. Only two questionnaires were found to be validated in other languages besides the original one. The content validity evaluation of UJACAS revealed its comprehensiveness in covering various aspects of AA. Specifically, five items mainly addressed one content area, while the others encompassed more than one content area. In terms of assessed aspects and translations, the UJACAS scale could be considered the most complete questionnaire for AA evaluation. Furthermore, recognizing the heterogeneity of the terms related to the aspects investigated could help professionals in selecting assessment tools with greater precision.

**Supplementary Information:**

The online version contains supplementary material available at 10.1007/s10433-025-00893-7.

## Introduction

### Conceptualization of active aging

The phenomenon of population aging is globally significant, with several factors contributing to this trend (e.g., longer life expectancy and greater quality of health care) (Kim et al. 2023). Consequently, as cohorts live longer and have fewer children, these factors have caused changes in the age structures of the population (United Nations 2020). According to the United Nations (2020), older persons are generally defined as such in relation to their chronological age, i.e., from 60 or 65 years of age onwards. Given this demographic shift, ensuring the well-being and autonomy of older individuals has become an important challenge in modern society, given the complexities associated with age-related health issues—cardiovascular, neurodegenerative, musculoskeletal and neuropsychiatric disorders (Li et al. 2021; Sathyanarayana Rao and Shaji 2007). For this reason, the need to preserve health, vitality and independence of older people currently represents a challenge of paramount importance (Storeng et al. 2018). Following this view, the perspective on aging has changed significantly in the gerontological literature. There was a shift away from a pathological perspective of later life to a positive perspective considering later life as a period of *success* and *productivity* (Johnson and Mutchler 2014). As a reflection of this positive perspective of aging, the concept of active aging (AA) was defined by World Health Organization (WHO 2002) as “*the process of optimizing opportunities for health, participation and security in order to enhance quality of life as people age”* (WHO 2002, p.12). Additionally, AA is a goal of health, and social policies aimed to improve three *pillars*: security, health and participation (WHO 2002). At practical level, AA is influenced by: (a) economic determinants (income capacity, income social protection, autonomy in managing finances, work activity); (b) availability of health and social services (access to healthcare services, to formal caregivers, support to informal caregivers); (c) behavioral determinants (tobacco and alcohol use, regular physical activity, nutritional habits, dental health, pharmacy); (d) social determinants (social support, protection from violence and abuse, literacy and learning); (e) physical environment (environmental characteristics, safe housing, public transport availability, falls, access to clean air and water, safe food); (f) personal determinants (e.g., cognitive functioning, mental health) (WHO 2002). Moreover, International Longevity Centre (2015) expanded the model adding a fourth pillar: *lifelong learning*. Recently, Hijas-Gómez et al. (2020) conducted a study with the aim of operationalizing the AA framework taking each pillar as an individual construct and using principal component analysis. According to the authors, each pillar is composed as follows:

- Health: psychological health, sleep health, physical health.
- Participation: social activities, social support and leisure perception, physical activity.
- Security: institutional support, leisure grants, housing issues.
- Lifelong learning: level of education and attendance at training activities.

It remains that a fundamental step for professionals involved in promoting aging care is to be able to use streamlined and effective measures to evaluate the quality of AA in older people.

### Evaluation of active aging

Evaluation of active aging (AA) is an important issue for policymakers as well as for professionals of care to promote evidence-based interventions improving older peoples’ quality of life (Han et al. 2023). At international level, the first quantitative measure of AA was the Active Aging Index (AAI) developed by Zaidi et al. (2013). AAI was calculated in 28 European Union (EU) countries, and it is composed by 22 indicators based on four domains: employment; participation in society; independent/healthy and secure living; capacity and enabling environment for active aging (i.e., remaining and healthy life expectancy, educational attainment). According to the authors, AAI is useful to develop policies and programs to deal challenges related to aging and it can distinguish multidimensional characteristics of AA. Considering this international perspective, index and sub-indices provide comparable data within EU countries related to untapped potential and various aspects of AA (i.e., social participation, independence, etc.). Calculation of AAI requires access to international surveys and databases. At individual level, AA could be measured through multiple self-report measures (for a review: Han et al. 2023). The assessment at individual level is also useful to analyze subjective well-being, perceived health, individual income and environmental resources (Barslund et al. 2017). Although international and individual level measurements of AA were distinguished, there is no universal consensus on how to measure it due to the presence of a wide range of models and indicators (Paul et al. 2012; Hijas-Gómez et al. 2020). A recent review conducted by Han et al. (2023) aimed to identify the most recommendable measurement tools of AA for use in clinical and research contexts. This was based on an assessment of the psychometric properties of the tools in question, as well as a quality assessment of studies using the Consensus-based standards for the selection of health measurement instruments (COSMIN) risk of bias methodology (Han et al. 2023; COSMIN 2022). Additionally, a scoping review was conducted by Xiao et al. (2024) with the aim to identify available tools for the evaluation of AA in older individuals and their defining characteristics (e.g., year, Country, theoretical framework, number of items, dimensions, sample size, reliability, validity and study level, i.e., individual or macro level) with a particular focus on the applications of these in China.

However, although it is known the presence of a wide range of models and indicators (Paul et al. 2012; Hijas-Gómez et al. 2020), this issue remains poorly investigated. Based on this evidence, it is reasonable to assume that the construction of measurement instruments may often reflect the heterogeneity in AA theories and indicators. As a result, the process of selection of an appropriate questionnaire based on the interest of the researcher and the assessment process itself could be longstanding. Additionally, a choice of questionnaire not in line with the research objectives could lead to an unfocused assessment process with the potential risk of burdening respondents, a major problem when respondents are older people (Rolstad et al. 2011). Indeed, older people reported lower rates of participation/less reliable responses in surveys than younger people due to physical (e.g., sensory disabilities), mental (e.g., cognitive functioning), and motivational (Motel-Klingebiel et al. 2014; Wagner et al. 2018) limitations.

The presence of several models and indicators of AA and the lack of a universal consensus on how to measure this construct could lead to confusion in choosing the most appropriate questionnaire for the assessment process. For this reason, identifying available questionnaires for the assessment of AA could be useful to help aging care professionals in selecting effective, fast and appropriate questionnaire based on the specific aspects of interest. Indeed, the selection of an appropriate questionnaire is crucial to lighten the assessment process and minimizing the burden on respondents by limiting the number of questionnaires they are required to complete, especially when respondents are older people (Rolstad et al. 2011). Additionally, the presence of different models/indicators and different questionnaires highlights the need to analyze in detail each instrument and the specific aspects of the construct investigated by each subscale and item of each instrument.

### Research design and purpose/aims

Bearing these issues in mind, our research is focused on the analysis of content areas such as the physical, social, economic, cognitive, psychological/emotional, ecological, and cultural ones and other characteristics such as aims of the questionnaire, available translation for a specific cultural context. Identifying these specific aspects for each questionnaire could be useful to get a comprehensive overview of the different indicators and aspects employed to assess the intended construct. At practical level, it could be useful in clinical and research contexts to help tool users during the choice and assessment processes.

The present research, developed in two studies, had the following specific aims:

1. To conduct a scoping review on available questionnaires for evaluation of AA and to analyze their contents (Study I). A scoping review is useful to identify potential size of available evidence and knowledge gaps, also involving ongoing research (Grant and Booth 2009; Mak and Thomas 2022). In particular, the Study I is focused on reporting the following information regarding each questionnaire: (a) content areas investigated and their adaptation to the AA determinants (WHO 2002; International Longevity Centre 2015), that is, physical, social, economic, cognitive, psychological/emotional, ecological and cultural areas and the related items/indicators, (b) main aims of the studies introducing the questionnaire and, (c) available validation information in any possible language and cultural context.
2. Based on results obtained from Study I, Study II performed a detailed content validity evaluation of a specific AA questionnaire deemed most complete based on number of covered areas (Almanasreh et al., 2019), number of available validation information and translations into different languages.

## Study I—Scoping review on measurement tools for the assessment of active aging

The aim of Study I was to conduct a scoping review to identify available questionnaires for evaluation of AA and to analyze respective assessed aspects.

### Methods

*Eligibility criteria* This scoping review was conducted in according to The Preferred Reporting Items for Systematic Reviews and Meta-Analyses Extension for Scoping Reviews (PRISMA-ScR; Tricco et al. 2018). PRISMA-ScR enables methodological quality to the study providing a checklist and flow chart to describe steps of source selection (Tricco et al. 2018).

The inclusion criteria were the following: (a) documents written in English, (b) articles published in journals and (c) gray literature. Gray literature also refers to studies that do not report significant findings and therefore are often not published in scientific journals (Paez 2017). For these reasons, gray literature is an important resource for literature reviews because it may reduce publication bias, and it may a more complete framework of evidence (Paez 2017; Haddaway and Bayliss 2015). Since the construct of AA was coined in 2002 (WHO 2002), (d) documents written from 2002 to 2025 were selected. We included (e) studies that presented a questionnaire for measuring AA for the first time. Additionally, (f) studies with adaptations and/or validations of the questionnaires in other languages were also specified. Exclusion criteria: secondary studies and documents not written in English were excluded.

*Search session* The search session was conducted on September 3rd, 2025. Scopus, Web of Sciences (WoS), PsycINFO, and PubMed databases were used to select eligible studies. Scopus and WoS include multidisciplinary coverage (Archambault et al. 2009). PsycINFO covers behavioral science and mental health areas, while PubMed collects documents of biomedicine and health literature (Goossen 2020). Since databases such as Scopus and WoS contain more articles published in journals (Mongeon and Paul-Hus 2016), Bielefeld Academic Search Engine (BASE) and OpenGrey databases were used for the search of gray literature. The search strategy was defined using the following query based on title, abstract and keywords of papers: “active aging” OR “active ageing” AND “measur*” OR “measurement tool*” OR “scale” OR “test*” OR “questionnaire*”. Queries modified and adapted for each database are presented in Supplementary materials (Supplementary Tables S1, S2). Other studies were found by the inspection of the reference lists of the selected papers.

**Fig. 1:**
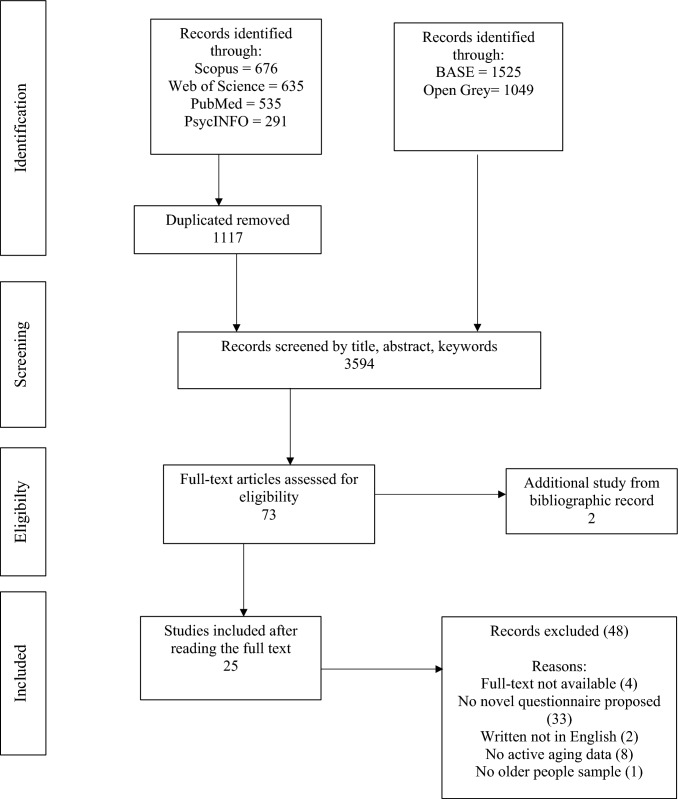
Flowchart of source selection

*Data extraction and article selection* From the selected records, duplicates were removed. Selected documents were screened for eligibility in two specific steps. In the first step, documents were assessed by title, abstract, and keywords to verify that they met the inclusion and exclusion criteria, i.e., proposal of a new questionnaire for active aging evaluation/validation of an existing questionnaire in another language. In the second step, full texts were analyzed for a further check following the aforementioned inclusion and exclusion criteria. For each included document, the following information was extracted: year, Country and language, sample age, study design (e.g., cross-sectional or longitudinal), investigated content areas, and aim of the questionnaire. The process of article selection was conducted by two independent raters (RN and GS). Additionally, a third independent rater (AL) was involved to solve disagreements, and the final decision was based on consensus among the three reviewers. A total of 25 documents were included in the final sample. In Fig. 1, the process for the inclusion of studies in accordance with the eligibility criteria is shown.

### Results

#### Summary of findings

Table 1 shows information of each included study, i.e., year of publication, Country, sample, and study design. Among them, 21 studies proposed a new questionnaire, and the remaining four were translation and adaptation studies. Table 2 shows main characteristics of each included questionnaire.

**Table 1:** Main characteristics of included studies

Questionnaire/Year	Country	Sample age	Study design
Active Aging Awareness Questionnaire (AAAQ) Ahmad Bahuri et al. (2021)	Malaysia	Three different samples, mean age (49.40; 49.90; 50.19)	Longitudinal
Active Aging Well-Being Index (AA-WB) Fritzell et al. (2021)	Sweden	> 60 years	Data from national surveys
Self-Aging Index (SAI) Gonçalves et al. (2017)	Portugal	≥ 55 years	Data from a national survey (Study of the Aging Profiles of the Portuguese Population, EPEPP) and a cross-national panel dataset (Survey of Health, Aging and Retirement in Europe, SHARE)
AAI—Bangladesh Haque and Afrin (2022)	Bangladesh	≥ 60 years	Cross-sectional
Active Aging Scale for Thai Adults (AAS—Thai) Thanakwang et al. (2014)	Thailand	≥ 60 years	Longitudinal
Vietnamese Active Aging Index (VAAI) Pham et al. (2020)	Vietnam	≥ 55 years	Longitudinal
Marsillas et al. (2017)	Spain	≥ 60 years	Cross-sectional
Oliveira and Tavares (2020)	Brazil	≥ 60 years	Cross-sectional
Paúl et al. (2012)	Portugal	≥ 55 years	Data from national surveys
Morales Ortiz and Fernandez (2020)	Spain	≥ 48 years	Cross-sectional
da Silva Sousa and De Azevedo Barros (2020)	Brazil	≥ 60 years	Cross-sectional
Taiwan Active Aging Index (TAAI) Hsu et al. (2019)	Taiwan	≥ 55 years	Cross-sectional
Yilmaz et al. (2023)	Turkey	> 60 years	Cross-sectional
Self-Active Aging Index (S-AAI) Keeratisiroj et al. (2023)	Thailand	≥ 60 years	Data from national surveys
Punyakaew et al. (2023)	Thailand	≥ 60 years	Longitudinal
Individual Active Aging Index for European Countries (AAI- EU) Barslund et al. (2017)	13 European Countries	≥ 55 years	Data from a cross-national panel dataset (Survey of Health, Aging and Retirement in Europe, SHARE)
The University of Jyvaskyla Active Aging Scale (UJACAS) Rantanen et al. (2019)	Finland	≥ 60 years	Longitudinal
Buys and Miller (2012)	Australia	≥ 50 years	Cross-sectional
Active Community Asiamah et al. (2020)	Ghana	≥ 60 years	Cross-sectional
Haque et al. (2016)	Thailand	≥ 60 years	Data from national surveys
Active Aging Scale—Urdu language (AAS-Pak) Bibi et al. (2023)	Pakistan	≥ 60 years	Longitudinal
University of Jyvaskyla Active Aging Scale (UJACAS)—Sweden language Nordeström et al. (2024)	Sweden	≥ 55 years	Longitudinal
University of Jyvaskyla Active Aging Scale (UJACAS) – Turkey language Erbil and Hazer (2019)	Turkey	≥ 60 years	Longitudinal
University of Jyvaskyla Active Aging Scale (UJACAS)– German language Hinrichs et al. (2024)	Switzerland	≥ 65 years	Longitudinal
Marsillas et al. (2025)	Spain	≥ 60 years	Cross-sectional

**Table 2:** Main characteristics of included questionnaires

Questionnaire	Original language	Term referring to the assessed aspects of AA				Covered content areas and related items				Aim	Other validation
			Physical	Psychological	Economic	Social	Cognitive	Cultural	Ecological		
Active Aging Awareness Questionnaire (AAAQ) Ahmad Bahuri et al. (2021)	Malay	/	–	–	–	–	1, 3: Awareness of specific elements of AA: 4: free from chronic disease 6: free from disabilities 7: regular exercise 9: healthy food 10: no alcoholic beverages 11: good oral health 12: healthy minds 14: not feeling lonely 16: participation in voluntary activities 17: participation in social activities 20: hobbies 21: learning new things 22: good amount of saving for retirement 23: own house	–	–	Monitoring awareness regarding AA in middle-aged people	/
Active Aging Well-Being Index (AA-WB) Fritzell et al. (2021)	Swedish	Domains	2.2: Physical activities 3.1: Mobility 3.2: able to perform ADL 3.4: Self-rated health 3.5: Life-expectancy at 75. 4.1:Access to health care 4.2:Access to dental care	3.3: Mental health	1.1: Cash-margin 1.2: No financial difficulties 1.3: Not at risk-of-poverty	**2.1: Cultural and leisure activities** 5.1: Cohabiting 5.2: Family contacts 5.3: Social contacts 6.1: Care for older people 6.2: Political participation 6.3: Activity in organization	6.5: Reading newspapers	**2.1:Cultural and leisure activities**	-	Comparing levels of AA to detect inequalities and changes over the time in oldest old	/
Self-Aging Index (SAI) Gonçalves et al. (2017)	Portuguese	Domains	Complaints about health status BMI Waist measure Falls (number, moment, cause, sequelae) Mobility (indoor, outdoor, climbing stairs) ADL e IADL Exercise (walk and sport) Smoking	Complaints about emotional status Feeling of dismay/ hopelessness Lack of interest Feeling nervousness/ anxiousness Sleep problems Lack of energy	Actual job	Social network Marital status Household size Time alone in 24 h period Confiding with someone	Spatial and time awareness **Gardening and** **Housekeeping**	–	**Gardening and** **Housekeeping**	Providing a self-assessment of AA status for older people	/
Active Aging Index (AAI)—Bangladesh Haque and Afrin (2022)	English	Dimensions	111–114: Sleep quality, walk, diet 201: Self-related health status 203: Disability 204: Treatment of disease 204(1): Regular medications 204(2–3): Local accessibility to health care 205: ADL 206: IADL 207: Alcohol drinking, smoking **302: Help in activities**	120: Satisfaction with life 202: Self-related mental health 304: Satisfaction with family services 407: Fear to be alone in house	110: Family income **115–116: Access to ICT** 119: Elderly friendly house 401: Income 402: Monthly income 403: Source of income 405: Home ownership 406: Elderly friendly housing environment	103: Marital status 104: Head of household **302: Help in activities** 303: Engagement in social activities	105: Education **115–116: Access to ICT** 118: Decision making 501–514: Perception about AA **117:** **Watching TV,** **listening to radio,** **reading newspaper**	**117:** **Watching TV,** **Listening to radio,** **reading newspaper**	–	Monitoring QoL in older people	/
Active Aging Scale for Thai Adults (AAS—Thai) Thanakwang et al. (2014)	Thai	Factors	1: Being self-reliant 5: Healthy lifestyle	3: Spiritual wisdom	4: Financial security	2:Engagement with society 7:Strengthening family ties to ensure care	**6: Engagement in active learning**	**6:Engagement in active learning**	–	Assessment of AA levels	Bibi et al., (2023)—Language: Urdu
Vietnamese Active Aging Index (VAAI) Pham et al. 2020	Vietnamese	Domains	–	WELL-BEING wb1: Happy or good mood wb2: Peaceful and relaxed wb3: Full of energy wb4: Feeling fresh and rested wb5: Daily life filled with things that interest me	BASIC NEEDS: mdprv3: Buy meat frequently mdprv2: Unexpected expense mdprv1: Utility bill INTERM NEEDS mdprv4: Traveling or tourism mdprv5: TV mdprv6: Fridge HIGH NEEDS: mdprv8: Washing machine mdprv7: Air conditioner mdprv9: Motorbike	VOLUNTARY ACTIVITIES vol1: Community and society services, **vol2: Cultural, educational, sport, occupational organizations,** vol3: Charity activities, vol4: Human rights, environment	–	**vol2: Cultural, educational, sport, occupational organizations,**	–	Measuring and tracking AA levels	/
Marsillas et al. (2017)	Spanish	Dimensions	PHY1: walk/ride PHY2: sport/exercise OH1: median symptoms (two weeks) OH2: chronic diseases SH1: performance ADL SH3: self-perceived health F2: ADL/IADL (Mahoney and Barthel 1965; Lawton and Brody 1969) **Art1: singing and playing instruments** **Art2: crafting** **Prod2: cooking**	OH3: psychological distress (three months) SL1: doing desirable things SL2: happiness to count on others SL3: feeling of energy last week SL4: feeling of confidence about future last week G1: stimulation in anything last week G2: satisfaction for achievement anything last week Emo1: having fun last week Emo2: feeling of happiness last week Emo3: feeling of euphoria last week SWL1: satisfaction related to ideal of life SWL2: conditions of life SWL3: satisfaction with life SWL4: obtaining important things SWL5: no changes in life EC3: expression of feelings AC3: being positive and learning from difficult situations AC4: taking difficult situations with humor **Sol1: spending time to be alone** **Sol2: hobbies**	E1: paid work (last week) **ICT1: using phone** **ICT2: using computer** **ICT3: using internet** **Sol2: hobbies** **Out1: going theater** Out2: traveling Recr1: watching TV	SP1: care children/ grandchildren SP2: care older people/disable SP3: unpaid voluntary activities (12 months) SP4: political activities (12 months) SA1: play cards with others (six months) SA2: visit others (six months) SA3: religious activity (six months) Soc1: meeting friends Soc2: receiving visits Soc3: counting on others Soc4: talking with someone Soc5: going out with people EC1: obtaining affect from others EC2: obtaining help from others Out3: being member of association EC3: expression of feelings Soc6: receiving help Soc7: receiving love and affect Soc8: receiving love from family **Out1: going theater**	**LL1: going to lectures in the last six months** **LL2: going to seminaries, courses** **LL3: reading books and newspapers** SH2: memory and attention affection Cog1: MEC-30 Lobo et al. (1999) AC1: changing situations AC2: solving problems AC3: being positive and learning from difficult situations **Art1: singing and playing instruments** **Art2: crafting** **ICT1: using phone** **ICT2: using computer** **ICT3: using internet** **Prod2: cooking** Recr2: Sudoku **Sol1: spending time to be alone**	**Out1: going theater** **LL1: going to lectures in the last six months** **LL2: going to seminaries, courses** **LL3: reading books and newspapers** **Sol2: hobbies**	Prod1: gardening	Evaluation of different elements of AA at the individual level	/
Oliveira and Tavares (2020)	Portuguese	Determinants	Functioning of hearing, vision, taste, smell, touch Morbidities Physical activity Physical security and protection Changes in health status Current health status Sleep quality Alcohol use Smoking Vaccination status Family history of CNCDs Access to health care Link with health services Preventive examinations Routine check-up **Physical environment (means of transport, home security)**	Resilience Depression/ emotional **Physical environment** (feeling safe)	Monthly income Economic condition Money to meet basic needs **Physical environment** (**means of transport, home security)** Environment in the home Retirements/ Pension Paid work	Social network Social support Personal relationship Community activities Out-of-school activities	Cognitive ability (MMSE, Bertolucci et al.^1994^)	–	**Physical environment** (climate, pollution)	Assessment of AA determinants	/
Paúl et al. (2012)	Portuguese	Determinants	Performing ADL e IADL Pulmonary function Strength Subjective health Illness Subjective physical activity Vision Audition Smoking **Sleep problems**	Psychological distress Happiness Personality Optimism Loneliness Life satisfaction **Sleep problems** Quality of life	Income	Social network	Cognitive functioning (MMSE, Bertolucci et al. 1994)	–	–	Assessment of AA determinants	/
Morales Ortiz and Fernandez (2020)	Spanish	**/**	**Crafting**	**Spending time in hobbies**	Autonomous management of finance **Using computer** **Spending time in hobbies** **Going to theater, watching film**	Going out with friends Taking part to discussions	Trying to learn new things **Using computer** Writing letters Listening to music **Going to theater, watching film Crafting Taking classes**	**Spending time in hobbies** **Going to theater, watching film** **Taking classes**	–	Assessment of cognitively stimulating activities	/
da Silva Sousa and De Azevedo Barros (2020)	Portuguese	/	Physical activity during leisure	Worship	Paid work Traveling **Use of internet**	Voluntary work/association **Sociocultural activities** Visits Personal contacts	**Access to regular and other courses** **Use of internet**	**Sociocultural activities** **Access to regular and other courses**	–	Assessment of engagement in activities	/
Taiwan Active Aging Index (TAAI) Hsu et al. (2019)	Chinese	Domains	3.1: Physical activity 3.2: Access to health and dental care **3.3: Independent living** 3.7:Physical safety from violence 3.9: Physical function independence 3.12: Primary prevention care utilization 3.13:Physical safety from injuries 4.1–2:Life expectancy **4.7:Transportation accessibility** **4.8: Transportation convenience** **4.9: Barrier-free space**	3.11: No depressive symptoms 4.3: Mental well-being	1:Employment 3.4:Relative median income 3.5: No poverty risk 3.6:No sever material deprivation 3.14: Owning assets **4.4:Use of ICT** **4.7:Transportation accessibility** **4.8:Transportation convenience** **4.9: Barrier-free space**	2.1: Voluntary activities 2.2: Care children/ grandchildren 2.3: Care disabled families 2.4: Political participation 2.5:Other social group participation 4.5: Social contact 4.10: Social integration and social respect	**3.3: Independent living** **3.8: Lifelong learning** 3.10: No severe cognitive impairment **4.4:Use of ICT**	**3.8:Lifelong learning**	**4.9:barrier-free space**	Monitoring AA and defining AA policies	/
Yilmaz et al. (2023)	Turkish	Dimensions	Taking long walks **Participating in sports activities** **Going to concert/** **watching film/** **playing instruments** **Repairing tools**	–	Traveling **Going to concert/** **watching film/** **playing instruments** **Repairing tools**	Talking to friend/relatives on the phone Meeting with friends/ relatives Volunteering activities Going to restaurants/cafes, etc. Participating to religious activities **Participating in sports activities** Participating in hobby groups	**Going to concert/** **watching film/** **playing instruments** **Repairing tools**	**Going to concert/** **watching film/** **playing instruments**	Gardening	Assessment social participation in older people which living in urban and rural areas	/
Self-Active Aging Index (S-AAI) Keeratisiroj et al. (2023)	Thai	Factors	Health1: Physical health Health 2: Visual Health3: Hearing Health 4: Barthel ADL (1965) Health 5: functional ability Health 6: Chronic diseases **Health 9: sleep problems** Health 11–12: oral care Health 13: BMI Health 14: physical activity Health 15: smoking Health 16: alcohol Health17: Annual check-up	Health 7: Psychological distress Health 8: No happiness **Health 9: Sleep problems**	Par1: Working Par3: Financial support to families Sec1: House ownership Sec2: Head of household Sec3: Residential safety Sec5: Income level Sec6: Source of income Sec7: Sufficiency of income Sec8: Saving bath Sec9: Debt bath	Par2: Marital status Par4: Member of group Par5: Participation in activities Par6: Meeting neighbors	Health 10: Forgetfulness problem	–	–	Assessment AA in older people which leaving in rural areas	/
Punyakaew et al. (2023)	Thai	Components	H1: Chronic condition H2: Pain and discomfort H3: Physical ability H5: Physical health condition **S2: Live conditions and Security**	H4: Mental health	S1: Financial stability **S2: Live conditions and Security** **E1: Use of ICT**	SP1: Religion SP2: Community SP3: Family and friends	**E1: Use of ICT** **E2: Literacy**	**E2: Literacy**	–	Assessment of AA levels	/
Individual Active Aging Index for European Countries (AAI- EU) Barslund et al. (2017)	English	Domains	3.1:Physical exercise 3.2:Access to health and dental care 3.3: Independent living arrangements 3.7: Physical safety from crime 4.1.-2:Remaining life expectancy	4.3: Mental wellbeing	1:Employment 3.4:Relative median income 3.5: No poverty risk for older person 3.6: No severe material deprivation	2.1: Voluntary activities 2.2: Care children/ grandchildren 2.3:Care to older adults 2.4: Political participation 4.5: Social connectedness	4.4: Use of ICT 4.6: Educational attainment **3.8: Lifelong learning**	**3.8: Lifelong learning**	–	Comparing levels of AA to detect inequalities	/
The University of Jyvaskyla Active Aging Scale (UJACAS) Rantanen et al. (2019)	Finnish	Sides	**1: crafting** **2: singing, playing instrument, drawing** **4: going out to nature** 5: physical exercise **8:help and support people** **14: improving coziness at home**	13: making days more interesting **14: improving coziness at home** 15: taking care of appearance 17: spirituality	**7: use of computer or iPad** 16. ensuring financial affairs	**1: crafting** **3: events (study and club/association)** **8: help and support people** 9:maintain social relationships 10: meet new people **12:taking responsibility to promote social and public questions**	**3: events (study and club/association)** **2: singing, playing instrument, drawing** 6:exercise mind and memory **7: use of computer or iPad** 11: taking responsibility to furthering life-related issues **12:Taking responsibility to promote social and public questions**	**3: events (study and club/association)** **12:taking responsibility to promote social and public questions**	**4: going out to nature**	Assessment of AA at individual level	Erbil and Hazer, (2019), Language: Turkish Nordeström et al. (2024), Language: Swedish Hinrichs et al. (2024), Language: German
Buys and Miller (2012)	English	Domains	F:Physical functioning, role limitations, bodily pain, general health, vitality G: Vision **H: Home and environment:** **access, visibility, safety, usability, maintenance, comfort**	E: Emotional well-being, Emotional and mental health D:Spiritual believes	A: Paid and voluntary work **H: Home and environment:** **access, visibility, safety, usability, maintenance, comfort**	C:Social: subjective support and social interactions	B:Learning new things	–	–	Measuring the three pillars of AA: health, security, participation	/
Active Community Asiamah et al. (2020)	English	Factors	HPS: health and paramedical services **Sas: sanitation and aesthetics** **SDCI: safety and disability friendliness of commercial infrastructure**	–	**SDCI: safety and disability friendliness of commercial infrastructure**	**SCOs: social centers and organizations** **PRSN: public services and road network** **SDCI: safety and disability friendliness of commercial infrastructure**	–	**SCOs: social centers and organizations**	**Sas: sanitation and aesthetics** **PRSN: public services and road network**	Monitoring the maturity of the built environment for social and physical activities	/
Haque et al. (2016)	Thai	Determinants	Exercise Oral health Medication ADL Subjective health Illness Visibility Hearing Falls Smoking Alcohol drinking Health promotion and disease prevention, Curative health services, Continuous care, Mental health care Annual health check-up **Location of bed** **Location of toilet**	Happiness level Psychological distress status	Work Income Sufficiency of income Savings **Location of bed** **Location of toilet**	Social support quality Marital status Community participation Participate in elderly group	–	–	–	Assessment of AA determinants and AA levels in older people	/
Marsillas et al. (2025)	Spanish	Dimensions	Independence in Basic Life Activities (ADL; Mahoney and Barthel 1965) Independence in Instrumental Life Activities (IADL; Lawton and Brody 1969) Presence of symptoms in the past two weeks Absence/presence of chronic disease Absence/presence of non-chronic disease or psychological stress in the past 3 months Perceived limitations in daily activities Perception of daily activity limited by cognitive state Satisfaction with health Perceived on health compared to peers **Singing/playing instruments Drawing or crafts** Walking Sports, exercise, or dancing **Gardening** Cooking	Satisfaction with relationship with neighbors Satisfaction with relationship with friends Satisfaction with help from family Satisfaction with time spent with family **Feeling loved by family** Feeling things are going well Feeling glad for having people to count on Feeling full of energy Feeling confident about the future Joy Cheer or happiness Euphoric **Interest Achievement**	Paid work **Use of mobile phone** **Use of computer** **Use of the Internet** **Cinema/Theater Traveling**	Frequency of contact with friends and family Visits Having people who care about oneself Possibility to talk to someone about problems Receiving invitations to entertain or going out Receiving help when being ill Receiving love and affection **Feeling loved by family** **Caring for children and grandchildren Political participation** **Volunteering** Playing cards/other games with people Visiting friends/relatives/neighbors	Mini-examen cognoscitivo Lobo et al. (1999) **Interest Achievement** **Caring for children and grandchildren** **Political participation** **Volunteering** Use of mobile phone Use of computer. Use of the Internet **Reading** **Use of mobile phone Use of computer** **Use of the Internet** **Games: Crosswords, Sudoku, etc**	Attendance to lectures. Attendance to courses within/outside the regular education system **Singing/playing instruments Drawing or crafts** **Cinema/Theater Traveling.** Associations or clubs TV watching Collect things	**Gardening**	Evaluation of AA at individual level	/

Note: - = content area not considered; / = validation studies not found; terms referring to assessed aspect of AA not specified; Items/indicators covering multiple areas are shown in bold

Abbreviation: AA = Active Aging; ADL = Activities of Daily Living; IADL: Instrumental Activities of Daily Living; QoL = Quality of Life; ICT = Information and Communication Technology; CNCDs = Chronic Non-Communicable Disease(s)

The following important features are reported for each questionnaire:

- terms used to refer to the assessed aspects of AA (e.g., “determinants,” “components,” etc.);
- content areas (e.g., physical, psychological, social, etc.) covered by items and number of items of the questionnaire, if specified;
- asserted aims underlying the development of the questionnaire;
- available translation / validation in other languages.

The first finding regarded different referring to the assessed aspects. Particularly, six studies used the term “domains” (i.e., Buys and Miller 2012; Gonçalves et al. 2017; Barslund et al. 2017; Hsu et al. 2019; Pham et al. 2020; Fritzell et al. 2021); three studies used “determinants” (Haque et al. 2016; Paúl et al. 2012; Oliveira and Tavares 2020); three studies used “factors” (Thanakwang et al. 2014; Keeratisiroj et al. 2023; Asiamah et al. 2020); four studies used “dimensions” (Marsillas et al. 2017; Haque and Afrin 2022; Yilmaz et al. 2023; Marsillas et al. 2025), one study used “components” (Punyakaew et al. 2023), and one study used “sides” (Rantanen et al. 2019).

Furthermore, often the generic term “health” or “personal factors” encompasses physical and mental health (e.g., Fritzell et al. 2021; Oliveira and Tavares 2020; Paúl et al. 2012; Hsu et al. 2019; Keeratisiroj et al. 2023; Punyakaew et al. 2023; Barslund et al. 2017). Most of questionnaires contained 4 or more subscales, except for the Active Aging Awareness Questionnaire (AAAQ; Ahmad Bahuri et al. 2021), Active Community, and two questionnaires developed by da Silva Sousa and de Azevedo Barros (2020) and Morales Ortiz and Fernandez (2020) which were not divided in subscales. Given this heterogeneity, it was chosen to group aspects using the term “content areas” to clarify specific investigated aspects:

- Physical area includes activities promoting mobility and physical efficiency, physical exercise, autonomy in performing activities of daily living;
- Cognitive area includes activities promoting perception, attention, learning, memory, thinking, decision-making, problem solving and language;
- Psychological/emotional area includes activities that promote psychological well-being (e.g., prevention of depression, anxiety, lack of energy, feelings of loneliness);
- Ecological area includes activities promoting the enjoyment of natural environments;
- Cultural area regards the participation in cultural activities;
- Economic area includes activities promoting financial autonomy, financial security, (light) work activity;
- Social area regards the participation in community activities, received/provided social support, preservation of social relationships (family and friends).

Analyzing the content areas covered by items, the questionnaires by Marsillas et al. (2017, 2025), the University of Jyvaskyla Active Aging Scale (UJACAS; Rantanen et al. 2019) and the Taiwan Active Aging Index (TAAI; Hsu et al. 2019) included items measuring all areas. On the other side AAAQ (Ahmad Bahuri et al. 2021) focused only on cognitive area. Generally, physical, psychological, social, cognitive, and economic areas were included in almost all the questionnaires while ecological and cultural aspects were less frequently considered. Moreover, 12 studies specified number of items. The two shortest questionnaires were composed by 11 items (da Silva Sousa and de Azevedo Barros 2020; Morales Ortiz and Fernandez 2020). On the contrary, one questionnaire had 177 items (Buys and Miller 2012) and the questionnaire UJACAS had 17 items for 4 subscales (that is, the same 17 content areas evaluated on four different aspects).

Regarding the aims of included studies, most of questionnaires aimed to assess levels of AA and the respective determinants. Particularly, three studies (Paúl et al. 2012; Oliveira and Tavares 2020; Haque et al., 2016) developed a new protocol for evaluation of AA determinants adapted from by other scales. Three questionnaires aimed to evaluate AA at individual level (Rantanen et al., 2019; Marsillas et al., 2017, 2025). On the other hand, AAAQ (Ahmad Bahuri et al. 2021) aimed to monitor awareness about AA, Activity Community (Asiamah et al. 2020) was developed to measure effective community characteristics to ensure social participation. The AAI for European Countries and AA-WB aimed to analyze inequalities of AA in older people (Barslund et al. 2017; Fritzell et al. 2021). The questionnaire of Morales Ortiz and Fernandez (2020) focused specifically on evaluation of cognitively stimulating activities and Self-Active Aging Index (S-AAI; Keeratisiroj et al. 2023) focused on older people living in rural areas.

In conclusion, only the Active Aging scale for Thai Adults (AAS-Thai; Thanakwang et al. 2014) and UJACAS (Rantanen et al. 2019) were validated and translated in other languages. Specifically, Bibi et al. (2023) validated the AAS-Thai in Urdu language and UJACAS were validated in Sweden, Turkish and German (Nordeström et al. 2024; Erbil and Hazer 2019; Hinrichs et al. 2024).

## Study II—Content validity evaluation of the University of Jyvaskyla Active Aging Scale (UJACAS)

The aim of Study II was to conduct a content validity evaluation of a chosen questionnaire for evaluation of AA. This could be useful to obtain a complete framework of evaluated aspects by a specific questionnaire and to ensure a greater dissemination for exhaustive evaluation of the construct.

Based on results by Study I, the UJACAS scale (Rantanen et al. 2019) was chosen for content validity evaluation in according to the following reasons:

- The scale covered all content areas. Consequently, this scale could detect the different aspects related to AA construct.
- Since this scale has a vast but not disproportionate number of items, it is reasonable to assume that UJACAS has the potential to assess appropriately aspects related to AA without increasing too much the burden on respondents. Additionally, it is possible to administer the scale as a questionnaire or an interview, this dual option allows the scale to be tailored to the needs of the respondent and/or the aim of the evaluator.
- To our knowledge, this scale was validated and translated in another three languages other than Finnish, of the Western European area i.e., Swedish, Turkish and German (Nordeström et al. 2024; Erbil and Hazer 2019; Hinrichs et al., 2024). Only another scale AAS—Thai (Thanakwang et al. 2014) was translated into another language (i.e., Urdu), and no other validation studies were found for the remaining questionnaires. A greater number of translations of an instrument could ensure cross-cultural comparisons (Bujang et al. 2022).

The UJACAS questionnaire is composed by 17 items corresponding to activities still carried out, usually, at a later age (e.g., social, physical, leisure, and cultural activities; management of finances and daily affairs; use of technological devices). Each item is developed into 4 subscales corresponding to the following questions:

- Goal: *“During the previous four weeks, I have wanted to…”.* This subscale refers to how strong was the desire to carry out activity.
- Ability: *“During the previous four weeks, I have or would have been able to…”.* This subscale refers to refers to how well the respondent was able to perform activity, based on health status and functional abilities (i.e., physical and mental abilities).
- Autonomy: *“During the previous four weeks, my opportunity to…”*. This subscale refers to the opportunity to carry out activity, considering matters related to life and environment.
- Activity: *“During the previous four weeks, I did…”.* Activity refers to the frequency of performing.

According to the authors, the four subscales reflect four central sides of AA, and each activity is assessed for the four sides. It was demonstrated that the questionnaire was able to assess a unidimensional latent construct of AA (Rantanen et al. 2019).

The total score ranges from 0 to 272 with higher scores indicating higher levels of AA.

Response scale selected is a 5 point Likert-like scale from 0 to 4. Response options range:

1. from “not at all” to “very strongly” for the subscale Goal;
2. from “not even with help” to “without any difficulties” for the subscale Ability;
3. from “It has not been possible” to “very good” for the subscale Autonomy;
4. from “not at all” to “daily or almost daily” for the subscale Activity.

According to the authors, the scale has good validity, reliability, and a test–retest reliability.

### Methods

Eight experts were identified among academics and scientists with expertise in the assessment of healthy and pathological aging. Literature suggested to use from five to ten raters to decrease probability of casual agreement (Almanasreh et al. 2019). In more detail, the experts were external to the research group and were selected according to two criteria: they were either PhD candidates or had a PhD in Psychometrics. All the experts gave their consent before proceeding. For each rater, an information kit and a response form (with items set at random) will be presented.

For each item, the asked question was “to which content areas do the activity listed in the item refer?”. Options were:

- 0 if the item does not refer to content area,
- 1 if the item does refer to content area.

It was also clarified that more than one content area can be indicated if the item was considered to cover more than one. The sums of the scores provided by the evaluators for each item and content area were calculated. To verify whether the items encompassed and assessed one or more content areas, the content areas that reported the first and second highest scores were identified. Subsequently, the ratio of the first and second highest scores was calculated. The term “content validity evaluation” was chosen to differ from the content validity analysis (e.g., Lawshe, 1975). Content validity analysis provides evidence that the components of an assessment are both relevant to and representative of the construct they are designed to measure (Almanasreh et al., 2019). In this case, we wanted to analyze how much each item on the UJACAS scale could cover one or more specific content areas identified for Study I.

### Results

Table 3 shows the sum scores for all eight raters. Specifically, each row corresponds to the sum scores for each item, while each column refers to the sum scores for each content area. The last column of the table shows the calculated ratio between the first and second highest scores related to content areas. The ratio scores allow us to analyze the extent to which each item covers one or more content areas. A ratio score of 2 or greater suggests that the item primarily addresses a single content area, corresponding to the content area that received the highest score. Conversely, a ratio score below 2 indicates that the item may encompass multiple content areas, corresponding to the content area that reported the first and the second highest scores.

**Table 3:** Sum scores of items and content areas of University of Jyvaskyla Active Aging Scale (UJACAS) based on eight raters

	Content areas							
item abbreviation	physical	economic	social	psychological/emotional	cognitive	ecological	cultural	ratio
1_crafting	7	1	0	2	5	0	1	1.4
2_artistic pursuit	3	2	0	4	6	0	3	1.5
3_events	2	1	7	3	2	1	7	1
4_nature	6	0	1	4	2	8	0	1.3
5_exercise	8	0	1	2	1	1	0	4
6_cognitive training	0	0	0	0	8	0	0	/
7_technology	2	1	1	1	6	0	2	3
8_help others	6	1	8	6	2	0	0	1.3
9_maintain relationships	1	0	8	5	1	1	0	1.6
10_meet new people	0	0	8	5	2	0	0	1.6
11_promote own matters	5	2	0	7	5	0	0	1.4
12_societal activity	0	0	7	4	2	1	5	1.4
13_make days interesting	0	2	0	7	3	0	3	2.3
14_make home cozy	0	1	2	7	2	1	0	3.5
15_appearance	3	3	2	5	1	0	1	1.7
16_economic balance	0	8	0	0	4	0	0	2
17_spirituality	1	1	6	4	2	3	5	1.2

Note: the items abbreviations referred to the University of Jyvaskyla Active Aging Scale. The content areas were physical, economic, social, psychological/emotional, cognitive, ecological, and cultural. The numerical values represent the total number of raters who assigned a particular item to a specific content area. For example, if five raters considered item 1 “crafting” pertained to the cognitive area, the score in that cell corresponds to 5. The last column reported the ratio between the highest and second-highest scores for each item. For example, the ratio for item 1 was 1.4 (corresponding to 7 divided by 5). In this case, the ratio was below 2, indicating that item 1 may encompass multiple content areas (i.e., physical and cognitive areas)

Overall, only item 6 (“cognitive training”) did not report ratio value because covered the cognitive area.

Ten items reported a ratio value less than 2. This indicates that the items could exhaustively cover more than one content area.

In particular, the cognitive area was covered by item 1 (“crafting”), item 2 (“artistic pursuit”), item 11 (“promote own matters”). The physical area was covered by item 1, item 4 (“nature”), item 8 (“help others"), item 11, and item 15 (“appearance”). The social area was covered by item 3 (“events), item 8, item 9 (“maintain relationships”), item 10 (“meet new people”), item 12 (“societal activity”) and item 17 (“spirituality”). Items 3, 12 and 17 covered also the cultural area. The psychological/emotional was covered by items 2, 8, 9, 10, 11, and 15. The economic area was covered by item 15 while the ecological area was covered only by item 4.

In conclusion, the remaining items reported a ratio greater than 2. This indicates that these items covered mainly one content area.

Item 7 (“technology”) and item 14 (“make home cozy”) covered the cognitive area while item 5 (“exercise”) covered the physical area. Item 16 (“economic balance”) covered the economic area and item 13 (“make days interesting”) covered the psychological/emotional area.

## General discussion

The general aim of the present studies was to identify existing questionnaires for the evaluation of AA to help health experts to choose faster the suitable questionnaire related to aim and to the aspects to be assessed. To realize this, a scoping review was conducted to identify existing questionnaires for the evaluation of active aging (AA) and to describe the characteristics related to assessed aspects, main aim and validations in other languages (Study I). Based on results obtained by Study I, a deepened content validity evaluation of the University of Jyvaskyla Active Aging Scale (UJACAS) was conducted to have a comprehensive framework of investigated aspects (Study II).

### Study I

In the present scoping review, 25 studies published between 2002 and 2025 were included. Main findings highlighted that the questionnaires are quite different in terms of covered areas by items and terms used to describe investigated aspects. Furthermore, it is worth mentioning that few studies presented validation in other languages of existing questionnaires.

At first, although most of the questionnaires were developed after 2015, only one study considered all four pillars of AA—security, participation, health, and lifelong learning—(da Silva Sousa and de Azevedo Barros 2020). Probably, authors referred to the AA model of the WHO (WHO 2002), rather than the expanded model that added lifelong learning as the fourth pillar (International Longevity Centre 2015). The addition of lifelong learning supports the other three pillars and underlines that learning plays an important role in improving well-being and quality of life in older people (Findsen and Formosa 2016). Future studies should refer to the updated model, considering all four pillars for validating new questionnaires.

Furthermore, while social, physical, psychological, cognitive and economic aspects were often considered in the AA evaluation process, ecological and cultural aspects were often overlooked. This could cause a loss of important information, since ecological and cultural aspects can significantly affect a successful and active aging. In the previous studies, it was demonstrated that green space accessibility predicted well-being and promoted physical and outdoor activities, social relationships, and participation in community (Lak et al. 2021; Zeng and Yang 2019; Sánchez-González and Egea-Jiménez 2021; Ricciardi et al. 2023; Spano et al. 2023). In this sense, green space accessibility becomes a resource to strengthen the four pillars of active aging.

Regarding cultural aspects, performing cultural activities (e.g., study groups) was related to a higher quality of life and satisfaction with health in older people (Koponen et al. 2023). Furthermore, the accessibility of cultural environments (e.g., exhibitions and museums) provides older individuals with the opportunity to engage in lifelong learning, which is the fourth pillar of AA. For these reasons, supporting cultural activities is important to support active aging policies (Hernández Lara and Toney 2021). Based on this evidence, the development of new questionnaires should insert items or indicators related to ecological aspects to analyze their influence on physical activities, social participation and perceived well-being.

Another important aspect concerns different terms used to present content areas. The terms “components”, “determinants”, “dimensions”, and “domains” are often used interchangeably.

The term “domains” often suggests broad, primary categories that encompass various aspects of a complex concept. In the context of AA, “domains” may refer to major areas of life (e.g., physical health, social engagement, psychological well-being) that collectively contribute to an individual's experience of aging. The use of “domains” implies a structured framework where each domain represents a distinct, yet interconnected, aspect of active aging. It reflects a comprehensive approach, treating each domain as integral to the whole. The use of “determinants” suggests a focus on the underlying factors or variables that influence AA outcomes. These are likely seen as causal agents or key drivers that shape how individuals age actively. Studies using “determinants” may emphasize the cause-effect relationships, such as socioeconomic factors, health behaviors, or environmental conditions that impact active aging. This term implies a more analytical or explanatory approach, concentrating on identifying and addressing root causes. “Components” implies a more *modular* or building-block approach, where different parts contribute to the whole. In AA, “components” might be specific elements (e.g., mobility, social participation, cognitive function) that together form the complete construct of active aging. The use of this term suggests that these elements can be individually analyzed or modified without necessarily altering the overall structure of active aging. It conveys a view of AA as an assembly of smaller, interdependent parts. The term “dimensions,” the most general one, suggests a multi-faceted, often qualitative view of AA, where different perspectives or axes of experience are considered. In the context of AA, “dimensions” might refer to different angles from which the aging process is examined, such as the subjective (e.g., self-perceived health), objective (e.g., functional abilities), or relational (e.g., social interactions) aspects. The use of “dimensions” indicates a flexible, layered understanding of AA, where each dimension adds depth and complexity to the overall concept.

These terms reflect differing theoretical perspectives and emphases—ranging from holistic and causal approaches to modular and multi-dimensional views of active aging.

This heterogeneity in used terms can reflect a lack of clarity in the AA theory. The model developed by WHO (2002) did not specify the difference between determinants and components of AA. Particularly, determinants such as financial security/economic factors and health were often defined as components, causing lack of clarity (Boudiny 2013). According to Boudiny (2013), the term “component” should be used to refer to an essential part of AA. On the other hand, health and financial security should be considered as instrumental resources to perform certain activities, rather than AA itself (e.g., economic finances and physical abilities might help to perform certain activities such as leisure or physical activities). Therefore, these aspects should be considered as determinants that can promote or hinder AA and not as components of AA (Boudiny 2013). However, there is still uncertainty about what the components of AA are and how to evaluate the construct (Belanger et al. 2017). Given this conceptual heterogeneity, highlighting the meanings of the different terms is important. On a practical level, clarifying these definitions can also provide benefits for professionals in clinical settings. Recognizing the specific function of each term could help professionals in selecting assessment tools with greater precision. For example, professionals might choose to evaluate a specific component (e.g., social participation) using a specific questionnaire, while evaluating a determinant (e.g., economic resources) with a different questionnaire, while recognizing heterogeneity and different meanings of the terms. This selective and tailored approach allows a personalized and refined assessment of the AA construct.

Five studies proposed new questionnaires for evaluation of AA adapted by other scales (Marsillas et al. 2017, 2025; Paúl et al. 2012; Buys and Miller 2012; Oliveira and Tavares 2020). Since the development of a new questionnaire requires rigorous steps, adapting instruments is a common process (Sousa et al. 2017). Given the multiplicity of questionnaires available, adapting existing questionnaires preserving their conceptual equivalence could be useful to reduce time and cost demanding actions (Hall et al. 2018; Sousa et al. 2017).

On the same line, we acknowledge that only two questionnaires (The University of Jyvaskyla Active Aging Scale, UJACAS; Rantanen et al. 2019; The Active Aging Scale for Thai Adults, AAS-Thai; Thanakwang et al. (2014) had validations in multiple languages. This represents a limitation in terms of study replication, comparative studies, and dissemination of the questionnaires. Adaptation and translation of questionnaires is an essential process to use them in new cultural and linguistic contexts (Gjersing et al. 2010). Moreover, cross-cultural adaptation of questionnaires is an important process to reduce risk of bias (Herdman et al. 1998) and allows a fair comparison between cultures of the same indicators (Bujang et al. 2022). For these reasons, future validations in other languages could be useful to ensure greater applicability of questionnaires. However, since most of the questionnaires were developed in 2020, it is reasonable to assume that the development of questionnaires and their respective validation studies in other languages is still at an early stage.

### Study II

Content validity evaluation was conducted identifying eight academics and scientists with expertise in the assessment of healthy and pathological aging. Particularly, it was calculated a ratio value to detect the coverage of content areas by each item.

At first, social relations and participation (i.e., items 3, 8, 9, 10) could encompass different factors in line with the literature. Previous evidence reported that enacted social support and participation in activities with formal support predicted life satisfaction and positive affect (Siedlecki et al. 2014; Blace 2012). Also, participation in activities with social support (e.g., assistance activities, carrying out errands for others) requires physical abilities (Blace 2012). Participation in cultural and social activities (e.g., taking part in art classes, going to theater), social support networks, and social integration, are associated with greater life satisfaction, positive affect, and affective balance (Reyes-Martínez et al. 2021). Cultural activities in older people are important to reduce stress and anxiety and to improve self-esteem (Murtin and Zanobetti 2024).

Regarding physical activity (i.e., item 5), psychological and emotional aspects are not dominant factors. This was explained in previous studies. Exercise has been shown in the literature to lead to improved well-being and life satisfaction, and physical activity could be considered a protective factor against depression (Ryu and Heo 2017; Kadariya et al. 2019). However, considering the intensity level of physical activity, a previous study showed that light physical activity (bowling, archery, electric trolley golf, fishing) was associated with life satisfaction and physical health, while moderate physical activity (e.g., light tennis, slow or light swimming, low-impact aerobics) was associated with physical health but not life satisfaction (Bae et al. 2017). For these reasons, although exercise may be associated with psychological well-being, this relationship is not always shown and may depend on the intensity of the exercise.

Moreover, leisure activities such as crafting and artistic pursuit (i.e., items 1, 2) could include the different aspects such as cognitive functioning and perceived well-being. In this regard, it was demonstrated that leisure activities predicted higher cognitive functioning and that cognitive functioning in turn predicted participation in leisure activities (Lifshitz-Vahav et al. 2017). Particularly, musical activity (e.g., dancing, playing instruments, and singing) is an important resource to promote well-being and cognitive health in older people (Dingle et al. 2021) and crafting activity , frequently performed in the elderly, enhances physical and mental health (Chacur et al. 2022).

Going outside and enjoying nature (i.e., item 4) could include aspects related to physical abilities and ecological characteristics in line with previous studies. Physical performance is an essential prerequisite to perform outdoor activities (Portegijs et al. 2014). On the other hand, it was shown that ecological characteristics (e.g., quality of air and near green space) could encourage older people to perform activities in natural environments (Yu et al. 2021).

Furthermore, using technology (i.e., item 7) comprises mainly cognitive aspects. In this regard, previous studies reported that problem solving ability, episodic memory and executive functioning were associated with technology devices (e.g., phone, computer, Internet) (Charness and Boot 2009; Choi et al. 2021). Other predictors were cultural knowledge and education. However, changes related to age in visual acuity (e.g., susceptibility to lightness) and in motor efficiency (e.g., arthritis) could hinder technology use. Considering this, although the utilization of technology is predominantly reliant on cognitive abilities, physical limitations may potentially obstruct the utilization.

In conclusion, the two items referred to activities to be performed independently (i.e., items 11, 16), include coherent aspects in according to the literature. Promoting own matters requires self-management abilities (e.g., positive affect, self-efficacy, taking initiative, investing in resources to obtain long-term benefits) which mediate the relationship between cognitive, physical functioning and well-being (Steverink et al. 2005; Cramm et al. 2013). Moreover, adequate cognitive and physical functioning ensure the maintenance of self-management abilities to preserve well-being (Cramm et al. 2013). Regarding financial balance, it was demonstrated that cognitive functioning and financial literacy may influence financial behaviors in older people (Kim et al. 2018). Particularly, worse cognitive abilities are associated with reduced self-efficacy which, in turn, decrease financial management ability (Tang 2021). Therefore, cognitive ability could be an important aspect of economic balance.

### Limitations

This work has limitations. The cross-sectional design adopted for many studies does not allow analysis of the test–retest reliability of the questionnaires. Another potential limitation may arise from the fact that only studies that had proposed the questionnaire considering a sample of older people were included. For this reason, some studies proposing a questionnaire for evaluation of AA may have been excluded.

On the other side, we believe that the greatest advantage of this work is represented by the practical potential for users of the questionnaires, both in clinical and research contexts.

Very frequently, the presence of different questionnaires for AA assessment and heterogeneous indicators could lead to confusion about the most appropriate questionnaire to use. Our work provides a valuable perspective for making informed and personalized choices when selecting a questionnaire. By identifying the areas each questionnaire covers and their respective validations in other languages, we offer a useful resource for evaluators, helping to streamline both the selection and evaluation process. Additionally, a targeted selection of questionnaires tailored to the evaluators' objectives would help reduce the burden on respondents.

## Conclusion

This work offers potentially valuable insights into the existing questionnaires for measuring AA, providing a practical resource for health experts. By identifying the specific content areas covered by each questionnaire and their validation status in different languages, this work aids in the informed selection of questionnaires, potentially reducing the time and burden associated with the evaluation process. Selected questionnaires are different in investigated aspects and each study uses different terms to describe these aspects: domains might be seen as broad, predefined categories for organizing knowledge about AA, determinants focus on causality, with an emphasis on factors that shape the process of active aging, components refer to individual elements that together constitute AA, suggesting a modular structure, dimensions represent various perspectives or axes of AA, offering a multi-faceted view of the concept. This suggests a potential confusion between what fundamentally constitutes AA and what factors influence it, likely stemming from a lack of conceptual clarity. Finally, this research could help aging care professionals toward a focused and informed selection of questionnaires according to their evaluation needs. Moreover, given that UJACAS scale covers the largest number of aspects related to active aging and is the most widely translated questionnaire in other languages, future validation studies could ensure greater diffusion of this questionnaire for an exhaustive assessment of active aging.

Future efforts should focus on enhancing the clarity of AA models, integrating ecological factors, and expanding the validation of questionnaires across various languages and cultures.

## Conflict of interests

The authors declare no competing interests.

## Supplementary Information

Below is the link to the electronic supplementary material.

Supplementary file1 (DOCX 22 KB)

## Data Availability

No datasets were generated or analyzed during the current study.
