# Supplementary material for: A scoping review of measurement tools and a content validity evaluation of the University of Jyvaskyla Active Aging Scale (UJACAS)
Source: Eur J Ageing. 2025 Nov 4;22(1):57. doi: 10.1007/s10433-025-00893-7 (PMC12586734; doi:10.1007/s10433-025-00893-7)
Supplement: Supplementary file 1 — Supplementary file1 (DOCX 22 KB) [file 10433_2025_893_MOESM1_ESM.docx]

**A scoping review of measurement tools and a content validity evaluation of the University of Jyvaskyla Aactive Aaging Sscale (UJACAS)**

European Journal of Ageing

Rosa Napoletano^1^, Antonella Lopez ^2^*, Sergio Traficante^1^, Elisabetta Ricciardi^3^, Luigi Tinella^4,1^, Alessandro Oronzo Caffò^1^, Andrea Bosco^1^, Giuseppina Spano^5^

**^1^** Department of Educational Sciences, Psychology, Communication, University of Bari, 70122, Italy

^2^ Department of Humanities, Social Science, and Education, University of Molise, 86100, Italy

^3^ Department of Precision and Regenerative Medicine and Ionian Area, University of Bari, 70124, Italy

^4^ Department of Humanities, Philosophy and Education, University of Salerno, Salerno, 84084, Italy

^5^ Department of Psychology and Health Science, Pegaso University, Naples, 80013, Italy

*Corresponding author:

Antonella Lopez, Department of Humanities, Social Science, and Education, University of Molise, Campobasso,

Via Francesco De Sanctis, 86100, Italy

E-mail address: antonella.lopez@unimol.it

**Table S1** Search query used in each database for searching measurement tools of Active Aging (AA) (updated on September 3, 2025)

| Database | Search query |
| --- | --- |
| Scopus | (TITLE-ABS-KEY (“active aging" OR "active ageing") AND TITLE-ABS-KEY ( "measur*" OR "measurement tool*" OR "scale" OR "test*” OR "questionnaire*” ) ) |
| Web of Science (WoS) | "active aging" or "active ageing" (Topic) and "measur*" or "measurement tool*" or "scale" or "test*" or "questionnaire*" (Topic) |
| PubMed | ("active aging" [Title/Abstract] OR "active ageing"[Title/Abstract]) AND (measur* [Title/Abstract] OR "measurement tool*" [Title/Abstract] OR scale* [Title/Abstract] OR test* [Title/Abstract]) OR questionnaire*[Title/Abstract]) |
| PsycINFO | ("active aging" OR "active ageing") AND (“measur*" OR "measurement tool*" OR "scale" OR "test*" OR "questionnaire*”) |

**Table S2** Search query used in each database of gray literature for searching measurement tools of Active Aging (AA) (updated on September 3, 2025)

| Database | Search query |
| --- | --- |
| OpenGrey  Bielefeld Academic Search Engine | "active aging" OR "active ageing" AND "measur*" OR "measurement tool*" OR "scale" OR "test*" OR "questionnaire*” |
| (BASE) | "active aging" OR "active ageing" AND "measur*" OR "measurement tool*" OR "scale" OR "test*" OR "questionnaire*” |
